# Explicit Action Switching Interferes with the Context-Specificity of Motor Memories in Older Adults

**DOI:** 10.3389/fnagi.2017.00040

**Published:** 2017-03-06

**Authors:** Carly J. Sombric, Harrison M. Harker, Patrick J. Sparto, Gelsy Torres-Oviedo

**Affiliations:** ^1^Department of Bioengineering, University of PittsburghPittsburgh, PA, USA; ^2^Department of Physical Therapy, University of PittsburghPittsburgh, PA, USA

**Keywords:** human, aging, generalization, locomotion, motor learning, set-shift, motor adaptation, split-belt treadmill

## Abstract

Healthy aging impairs the ability to adapt movements to novel situations and to switch choices according to the context in cognitive tasks, indicating resistance to changes in motor and cognitive behaviors. Here we examined if this lack of “flexibility” in old subjects observed in motor and cognitive domains were related. To this end, we evaluated subjects' performance in a motor task that required switching walking patterns and its relation to performance in a cognitive switching task. Specifically, a group of old (>73 years old) and young subjects learned a new locomotor pattern on a split-belt treadmill, which drives the legs at different speeds. In both groups, we assessed the ability to disengage the walking pattern learned on the treadmill when walking overground. Then, we determined if this motor context-specificity was related to subjects' cognitive ability to switch actions in a set-shift task. Motor and cognitive behaviors were tested twice on separate visits to determine if age-related differences were maintained with exposure. Consistent with previous studies, we found that old adults adapted slower and had deficits in retention. Most importantly, we found that older subjects could not switch locomotor patterns when transitioning across walking contexts. Interestingly, cognitive switching performance was inversely related to subjects' ability to switch walking patterns. Thus, cognitive mediated switching interfered with locomotor switching. These findings were maintained across testing sessions. Our results suggest that distinct neural substrates mediate motor and cognitive action selection, and that these processes interfere with each other as we age.

## Introduction

The motor system has the ability to develop and select context-specific locomotor memories, which enable us to navigate and transition between different terrains without falling. Consider that when walking on ice, we develop a context-specific gait that maximizes stability. We transition out of this gait when switching to a different terrain because it would be inefficient to walk the same way when movement demands change. While motor adaptation allows us to match environmental demands, it is faster to recall context-specific motor memories for the situation at hand (Shadmehr and Brashers-krug, 1997; Wolpert and Kawato, 1998). Therefore, developing and switching between context-specific motor memories enable us to immediately perform well as task demands change. Here we ask whether this critical ability to learn context-specific motor memories and switch between them is affected by healthy aging.

While it is well established that aging impairs learning of new motor patterns, it is unclear if it also affects the context-specificity of learned movements. Several studies in motor adaptation have shown that the rate at which people learn (McNay and Willingham, 1998; Fernández-Ruiz et al., 2000; Buch et al., 2003; Bock, 2005; Rodrigue et al., 2005; Heuer and Hegele, 2008, 2011; Hegele and Heuer, 2010, 2013; Anguera et al., 2011; Bruijn et al., 2012; Trewartha et al., 2014) and the final adapted state they reach are impaired with aging (McNay and Willingham, 1998; Seidler, 2006; Langan and Seidler, 2011; Bruijn et al., 2012; Huang and Ahmed, 2014). While there is a consensus that healthy aging impairs subjects' performance during motor adaptation tasks, it is unclear whether aging also affects transitioning between different context-specific motor memories. Previous work has shown that older adults transfer movement patterns learned in one situation to another when it is beneficial to performance (Bock, 2005; Bock and Girgenrath, 2006; Langan and Seidler, 2011; Wang et al., 2011) However, it is unknown if they also transfer information across situations when it is detrimental to do so. Thus, we will test whether older adults can disengage movements learned on a treadmill when they impair their performance in a different walking context.

We hypothesize that processes for cognitive switching contribute to motor switching in older adults. This is formulated on the basis of other studies showing that motor performance is influenced by cognitive abilities relevant to the motor task. For example, diminished spatial working memory in older adults decreases the ability to counteract visual (Anguera et al., 2011; Langan and Seidler, 2011) and force perturbations (Trewartha et al., 2014), and to learn spatial motor sequences (Bo et al., 2009). In addition, a recent study showed that interventions improving motor switching also improve cognitive switching (Coubard et al., 2011). Thus, we reasoned that cognitive and motor action selection might be related such that the cognitive ability to explicitly switch actions might contribute to transitioning between locomotor patterns when the environment changes. While it is well-known that older adults have limited ability for switching actions in cognitive tasks (Kramer et al., 1994; Klein et al., 2000; Van Asselen and Ridderinkhof, 2000; Friedman et al., 2007; Adrover-Roig and Barceló, 2010), it is unknown if this is correlated with difficulties switching patterns in motor tasks. Here we will test the extent to which age-related cognitive impairments for task switching is linked to age-related deficits in motor switching.

In sum, growing evidence indicates that cognitive and motor processes interact in motor learning, but little is known about their relation in action selection and its changes with healthy aging. Thus, we investigated age-related changes in context-specificity of locomotor learning and its correlation to deficits in cognitive switching. We predicted that cognitive switching would contribute to switching locomotor patterns when transitioning across different walking contexts. Conversely, we found that cognitive strategies for choosing actions interfered with locomotor switching in older adults. This suggests that cognitive-mediated processes for action selection impair, rather than compensate, for age-related deficits in motor switching.

## Methods

We investigated how healthy aging affects subjects' locomotor learning on a split-belt treadmill and its transfer to overground walking. We also tested if cognitive switching in older adults was related to the ability to switch locomotor patterns across walking contexts (i.e., treadmill vs. overground). To this end, 11 young adults (6 men and 5 women: 25.4 ± 5.5 years old) and 11 old adults (7 men and 4 women: 77.2 ± 2.8 years old) participated in the locomotor and cognitive tasks described below. We also evaluated if age-related differences were maintained after repeated exposure. Thus, eight young adults (4 men and 4 women: 27.0 ± 5.7 years old) and eight older adults (6 men and 2 women: 76.6 ± 2.5 years old) were tested again 6 weeks after their first visit. The Institutional Review Board at the University of Pittsburgh approved the experimental protocol and all subjects gave informed consent prior to testing.

### General paradigm

#### Locomotor task

**Figure 1:**
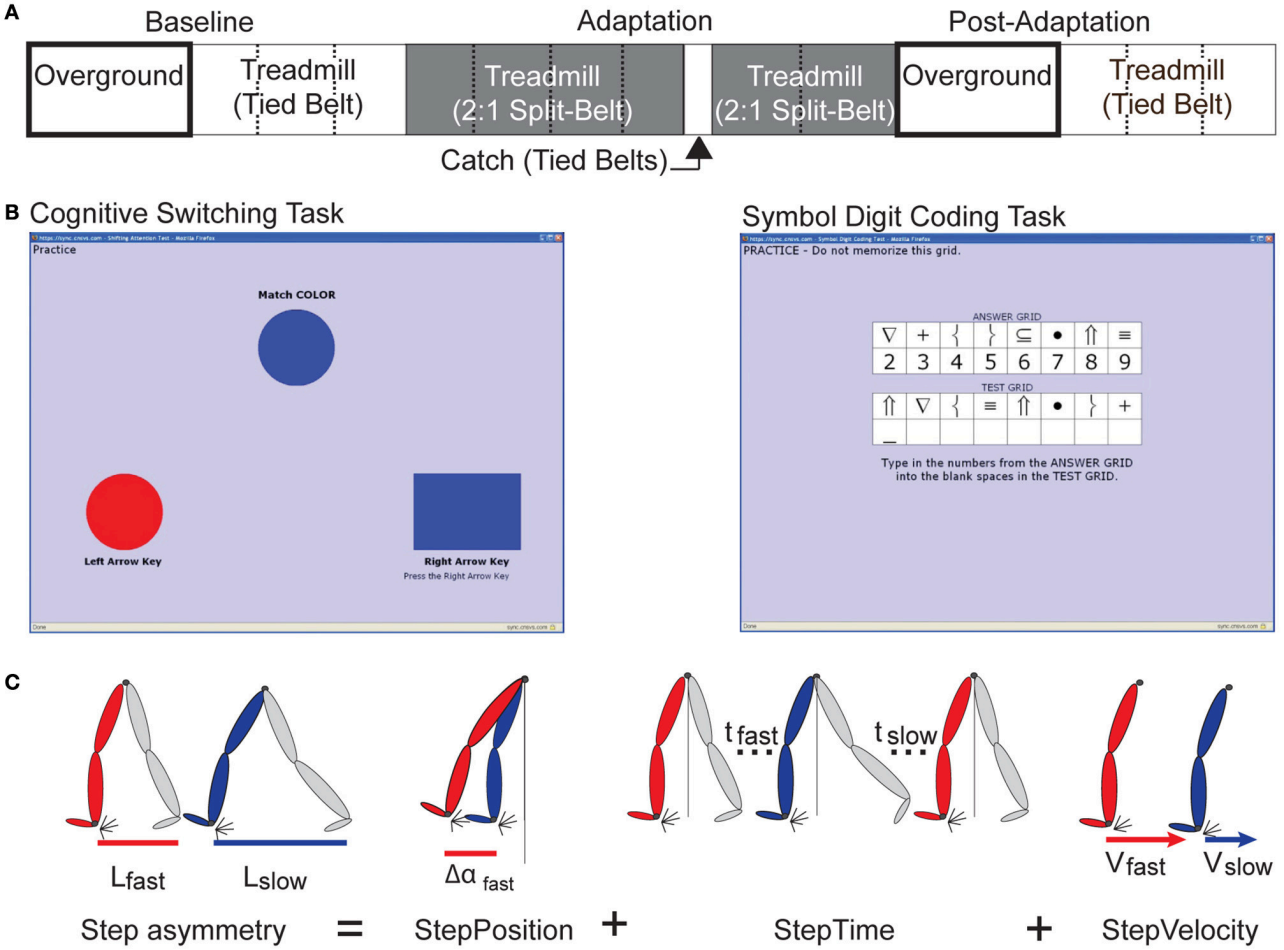
**Experimental Paradigms and Definition of Parameters. (A)** Here the split-belt treadmill paradigm used for both age groups during their first and second visits is illustrated. Resting breaks, when subjects did not walk, are indicated by dashed lines. These were taken every 150 strides. **(B)** The left panel is a sample screen for the cognitive switching task that was used to assess cognitive switching ability. The right panel is a sample screen for the Symbol Digit Coding Task, which was used to assess processing speed. **(C)** This schematic adapted from Finley et al. (2015) illustrates Step Length Asymmetry (StepAsym), StepPosition, StepTime, and StepVelocity parameters.

Adaptation and transfer of split-belt walking was assessed in all participants following the paradigm illustrated in Figure 1A. All subjects walked overground and on a treadmill during a baseline period. For overground walking, subjects walked back and forth on a 9.2 m walkway at a self-selected speed for 6 min (~150 strides). For treadmill walking, subjects walked at slow (0.50 m/s), fast (1.00 m/s), and medium (0.75 m/s) speeds for 150 strides each. A stride was defined as the time between two consecutive foot landings (i.e., heel-strikes) of the same leg. Then, subjects experienced an adaptation period for 2 blocks on a split-belt treadmill when the non-dominant leg moved at 0.50 m/s and the dominant leg moved at 1.00 m/s. Leg dominance was assigned based on the reported leg used to kick a ball. A catch period (10 strides) during which belts moved at the same speed (0.75 m/s) was administered after the first adaptation block of 600 strides. The behavior during this catch period was used to assess the amount of learning in the treadmill context. The second adaptation block of 300 strides was used to re-adapt subjects' gait, which was disrupted by the catch. Directly following the second adaptation block, all participants experienced a post-adaptation period overground and on the treadmill. During the post-adaptation period overground, subjects again walked back and forth on a walkway at their self-selected speed for 6 min (~150 strides). This period was used to evaluate the transfer of treadmill adaptation effects to a different walking situation. Subjects were transported from the treadmill to the walkway with a wheelchair to ensure the first steps after adaptation were recorded. Remaining context-specific after-effects were assessed during a post-adaptation period on the treadmill when subjects walked for 450 strides at a medium speed (0.75 m/s). When walking on the treadmill, all subjects took resting breaks every 150 strides during which they were not walking. On average older adults took breaks of 4.5 ± 1.5 min and young adults took breaks of 2.9 ± 0.7 min.

#### Cognitive task

Two cognitive abilities were evaluated: cognitive switching and processing speed. The assessment of these distinct cognitive abilities allowed us to determine if context-specificity of motor learning was generally related to overall cognitive capacity or to a specific cognitive ability. We tested cognitive switching because we hypothesized it would be correlated to the ability to switch locomotor patterns across walking contexts (i.e., treadmill vs. overground). We also tested processing speed because it has been shown to be correlated to walking performance in older adults (Chen et al., 2012; Odonkor et al., 2013) and motor adaptation in young subjects (Rodrigue et al., 2005). Cognitive switching was assessed with a Cognitive Switching Task (i.e., a set-shift task) and processing speed was evaluated with a Symbol Digit Coding Task (Figure 1B), as in previous studies characterizing age-related changes in these two cognitive functions switching (Gualtieri and Johnson, 2008; Klouda et al., 2017). In the Cognitive Switching Task, subjects had to match two objects based on randomly changing rules that were explicitly given to the subjects (i.e., “Match by color” or “Match by shape”) (Figure 1B, left panel). Participants were instructed to answer correctly and as fast as possible. They were given 2-s to answer at every trial during the task. Responses past the 2-s window were considered wrong. Participants performed the Cognitive Switching Task for 90 s, yielding at least 45 trials. In the Symbol Digit Coding Task, subjects had to match symbols to numbers based on a reference symbol-digit table. They were instructed to complete as many matches as possible within 2 min. Cognitive tests were performed in a quiet room with no distractions. The proctor provided verbal and written instructions and supervised practice trials prior to testing to ensure participants understood the tasks. Older adults took the cognitive assessments on both their first and second day, whereas younger adults only took the cognitive task on their first day. Note that cognitive tests of two older adults were excluded from the analysis because they were performing at chance levels and the cognitive test of one young adult was not recorded due to technical difficulties. Thus, our analyses were performed using 27 cognitive data points.

### Data collection

#### Locomotor task

Kinematic data were collected to characterize subjects' behaviors. Kinematic data were collected at 100 Hz with a passive motion analysis system (Vicon Motion Systems, Oxford UK). Gaps in raw kinematic data due to marker occlusions were filled with a quintic spline interpolation (Woltring; Vicon Nexus Software, Oxford Uk). Subjects' movements were assessed through passive reflective markers placed bilaterally on bony landmarks at the ankle (i.e., lateral malleolus) and the hip (i.e., greater trochanter). Markers were also placed asymmetrically on shanks and thighs to differentiate between the legs. Heel strikes, defined as the times when the feet landed on the ground, were identified with kinematic data. This was done to have equivalent event detection on the treadmill and overground as in previous transfer studies (Torres-Oviedo and Bastian, 2010, 2012).

#### Cognitive task

Subjects' cognitive switching and processing speed were assessed with CNS Vital Signs software (CNS Vital Signs, Morrisville, NC). Cognitive switching and processing speed were evaluated with the tasks called “Shifting Attention Test” and “Symbol Digit Coding Test” in the CNS Vital Signs software, respectively. The number of correct and incorrect responses were recorded in both tests. These tests on the CNS Vital Signs software have been validated as compared to conventional neuropsychological tests (Gualtieri and Johnson, 2006).

### Data analysis

#### Gait parameters for locomotor task

We assessed the behavior of spatial and temporal features of gait, which have been shown to adapt (Malone and Bastian, 2010; Malone et al., 2012; Finley et al., 2015) and transfer differently (Torres-Oviedo and Bastian, 2010). For a robust measure of adaptation we looked at step length asymmetry (StepAsym). This measure is conventionally used to characterize gait adaptation in split-belt studies (e.g., Reisman et al., 2005). StepAsym was defined as the difference in step lengths when taking a step with one leg vs. the other (where step length was the distance between ankles at heel strike). Thus, a zero value for StepAsym indicated that both steps were the same length. By convention, StepAsym is positive when the step length of the fast (dominant) leg is longer than the one of the slow (non-dominant) leg. The adaptation of StepAsym has been shown to be influenced by spatial and temporal gait features (Malone and Bastian, 2010; Malone et al., 2012; Finley et al., 2015). Therefore, to more specifically characterize gait adaptation, step length asymmetry was further decomposed into spatial (StepPosition), temporal (StepTime), and velocity (StepVelocity) components for two consecutive steps, as done previously (Finley et al., 2015, Figure 1C; Equation 1). These parameters represent distinct aspects of gait when taking a step with one leg vs. the other. Specifically, StepPosition quantified the difference in positions of the leading leg (i.e., leg in front of the body) between two consecutive steps (Equation 2). StepTime quantified the difference in the duration of each of these steps (Equation 3). Lastly, StepVelocity quantified the difference in the velocities of each foot with respect to the body for these two steps (Equation 4). We scaled the differences in step time and step velocity so that all parameters were in the same units of distance. This allowed direct comparisons across parameters. For visualization purposes, these parameters are smoothed with a 5-stride running average.

(1) $$\begin{array}{rcl} StepAsym & = & \frac{Fast\;Step\;Length-Slow\;Step\;Length}{SL} \end{array}$$

(2) $$\begin{array}{rcl} =\;StepPosition+StepTime+StepVelocity\\ stepPosition & = & \frac{\left(\Delta \alpha_{fast}-\Delta \alpha_{slow}\right)}{SL} \end{array}$$

(3) $$\begin{array}{rcl} StepTime & = & \;\frac{\frac{v_{slow}+\;v_{fast}}{2}\left(t_{slow}-t_{fast}\right)}{SL} \end{array}$$

(4) $$\begin{array}{rcl} StepVelocity & = & \frac{\frac{t_{slow}+t_{fast}}{2}\left(v_{slow}-\;v_{fast}\right)}{SL} \end{array}$$

In these formulas, Δα_*i*_ is a length measure that indicates the difference between each leg's landing position with respect to the body (index i represents the leg); *t*_*i*_ represents the step time defined as the duration between heel-strikes of one leg vs. the other; and v_*i*_ represents the step velocity quantified as the relative velocity of the body with respect to the ankle in contact with the ground. When walking on the treadmill, v_*slow*_ and v_*fast*_ approximate the speeds of the slow and fast belt, respectively. When walking overground, v_*slow*_ and v_*fast*_ are approximately zero. Therefore, StepVelocity directly represents the environment features, rather than subjects' behavior. Note that all measures were normalized by each subject's stride length (SL) to account for inter-subject differences in step sizes. Stride length was equal to the sum of two consecutive step lengths.

#### Outcome measures for locomotor task

Outcome measures were defined to characterize the adaptation, forgetting, learning, transfer, and washout of movements in the locomotor task. These measures were computed for each of the parameters defined above (StepAsym, StepPosition, and StepTime). A summary of locomotor outcome measures, their computation, and meaning is included in Table 1.

**Table 1:** **Locomotor Outcome Measures**.

**Outcome measure**	**Meaning**	**Calculation**
Steady State (*SS*)	Steady state of adapted movements at the end of split-belt walking	Mean value of the last 50 strides of split-belt walking
Extent of Adaptation (AdaptExtent)	Extent of adaptation to recover step symmetry in the split-belt environment	*AdaptExtent* = *SS*_*Sasym*_ − *SS*_*Sv*_
Time Constant (τ)	Rate at which each a gait parameter is adapted	Number of strides to reach 63.2% of steady state
%Forgetting	Average decay of adapted movements due to the passage of time	$\%Forgetting=\frac{1}{3}\sum_{i=1}^{3}\left(\frac{F_{i}-I_{i}}{F_{i}}\times 100\right)$
Learning index	After-effects due to newly acquired movements in the split-belt environment	Mean value of first 3 strides of the catch trial following split-belt walking
Transfer index	After-effects due to carry over of adapted movements to overground walking	Mean value of the first 5 strides of overground walking following split-belt walking
%Transfer	After-effects overground expressed as a percent of the extent of adaptation	Transfer index/AdaptExtent
Washout	Remaining after-effects following de-adaptation when walking overground	Mean value of the first 5 strides on the treadmill following overground walking
%Washout	Remaining after-effects expressed as a percent of the extent of adaptation	Washout/ AdaptExtent

*This table includes a comprehensive list of the outcome measures that were used, their meaning, and how they were computed*.

Adaptation was evaluated with three outcome measures: extent of adaptation, steady state, and time constant. The extent of adaptation (AdaptExtent) characterized how well subjects counteracted the split-belt perturbation. This outcome measure was computed as the difference between the steady state of StepAsym, which is a good proxy for subjects' adaptation (Reisman et al., 2005), and the steady state of StepVelocity, which is a good proxy for the split-belt perturbation (Finley et al., 2015) (Equation 5). In addition, we calculated the steady states of StepPosition (spatial) and StepTime (temporal) to determine if subjects had a preference for a spatial vs. temporal strategy to counteract the split-belt perturbation. Steady states for all parameters were computed as the mean value across the last 50 strides of the adaptation period (i.e., last 50 strides before walking overground) compared to the mean baseline behavior on the treadmill. Moreover, a time constant, τ, was calculated to evaluate the rate at which subjects adapted. τ was quantified as the number of strides that subjects took to reach 63.2% of their steady state. This time point was identified after smoothing each subject's adaptation curve with a 20-step running average, which we selected based on prior studies assessing adaptation rates during split-belt walking (Malone and Bastian, 2010; Vasudevan et al., 2011). As in such studies, the smoothing was done to prevent premature identification of the number of steps to reach 63.2% of the steady state behavior. Larger τ values indicated that subjects adapted slower than subjects with smaller τ values, and vice versa.

(5) $$\begin{array}{r} AdaptExtent=SS_{Sasym}-SS_{Sv} \end{array}$$

Forgetting was characterized with a %Forgetting measure. This measure indicated the effect of the passage of time during resting breaks on subjects' adapted motor state. Large %Forgetting values indicated that subject's motor memory decayed during the resting breaks whereas small values indicated that subjects maintained the adapted motor memory during the breaks. %Forgetting was quantified by computing the mean difference in adaptation levels before and after each break. To compute this measure, we first calculated the differences in adaptation levels before (F_*i*_) and after (I_*i*_) the ith break and then expressed each of them as a percentage of the adapted level that was reached before the corresponding break. Then an averaged %Forgetting was computed using the behavior before and after the first 3 breaks because there were no-significant differences in forgetting across these three breaks. The breaks after the catch trial were not included in the analysis because of the possible de-adaptation during tied walking in the catch trial. Formally expressed:

(6) $$\begin{array}{r} \%Forgetting=\frac{1}{3}\sum_{i=1}^{3}\left(\frac{F_{i}-I_{i}}{F_{i}}\times 100\right) \end{array}$$

Note that StepAsym approached zero as subjects adapted. Thus, τ and %Forgetting were not numerically robust when using the steady state of StepAsym to calculate them. Consequently, to compute them we used the steady state of StepAsym shifted by the perturbation each subject experienced, which was characterized by the steady state of StepVelocity (Finley et al., 2015). This shifted version of the steady state of StepAsym is equivalent to the extent of adaptation (Equation 5).

Learning was characterized with a learning index indicating the difference in behavior on the treadmill before and after adaptation. This was computed as the difference between the first three strides during the catch trial, when the belts moved at the same speed, and the averaged values across the last 50 strides of the baseline period before adaptation.

Transfer was characterized by the after-effects observed overground following split-belt adaptation on the treadmill. Large numbers indicated that subjects did not disengage the pattern learned on the treadmill when transitioning to a different walking context. The transfer index was calculated as the averaged difference between the initial 5 strides during overground walking directly following adaptation and the baseline behavior overground. The difference in overground walking before and after adaptation was calculated as a function of subjects' position on the walkway. By comparing post-adaptation to baseline in this manner, we were able to remove the effect of asymmetries associated with changes in walking velocity at the beginning and at the end of each pass on the walkway. In addition, all steps were systematically reviewed to remove asymmetries associated with making a turn at the end of the walkway. These two procedures allowed us to quantify asymmetries overground that were only due to split-belt adaptation, rather than those due to turning, starting, or stopping. Importantly, the conclusions from our results were not dependent on these procedures to assess baseline walking overground. Transfer was also expressed as a percent of AdaptExtent indicating how well subjects adapted on the treadmill. Different from previous studies (Torres-Oviedo and Bastian, 2010, 2012), we chose to normalize Transfer by this value, as opposed to the learning index, because it is one single value that indicates the maximum adapted state that StepPosition and StepTime could have reached. This normalization has two advantages. First it maintains the relation expressed in Equation 1 for %Transfer. Second, it indicates the carry-over to overground walking of spatial and temporal patterns used on the treadmill to counteract the split-belt perturbation.

Lastly, washout indicated the extent to which overground walking washed out the movements adapted on the treadmill. It was quantified by the remaining after-effects during the first 5 strides during the post-adaptation period on the treadmill compared to baseline treadmill walking. Similar to transfer, washout was also expressed as a percent of AdaptExtent (i.e., %Washout). Thus, 100% values indicated that 100% of the adapted state on the treadmill remained after overground walking.

#### Outcome measures for cognitive task

Cognitive switching ability and processing speed were assessed in all subjects. Cognitive switching ability was determined with an accuracy measure used to evaluate subjects' cognitive ability to switch actions according to the context. This was quantified with a unitless ratio of correct responses over total responses during the Cognitive Switching Task, as indicated in Equation 7. Processing speed was a measure used to represent subjects' speed to process information. It was quantified with a ratio of correct responses (i.e., correct symbol-number matches) over the 2-min duration of the Symbol Digit Coding Test (Equation 8). The 2-min period was converted to seconds such that processing speed had the units of number of correct matches per second.

(7) $$\begin{array}{r} \text{Cognitive Switching}=\frac{\#Correct\;}{\#Total} \end{array}$$

(8) $$\begin{array}{r} Processing\;Speed=\frac{\#Correct}{120\;sec} \end{array}$$

### Statistical analysis

An unbalanced two-way ANOVA was used to test the effects of exposure (i.e., first vs. second visits) and age group (i.e., older vs. young adults) on each of our outcome measures (e.g., τ, %Forgetting, etc.). This was done to account for the different number of participants on the first and second visits. All interaction terms were not significant. While all reported effects of age and/or experimental exposure were drawn from main effects of the two-way ANOVAs, we used Fisher's LSD *post-hoc* testing to assess if main effects were driven by individual test groups. To test if %Forgetting was a predictor of the rate at which subjects adapted (τ), we performed a multiple regression analysis to determine the predictive power of age group, exposure, and %Forgetting on τ. To determine if motor switching, quantified by Transfer and %Transfer, was related to cognitive abilities, we performed a linear regression analyses between each cognitive ability (i.e., cognitive switching and processing speed) vs. Transfer and %Transfer. Young and old groups were analyzed separately. A significance level α = 0.05 was used for all analysis. Stata was used to perform all statistical analysis (StataCorp LP, College Station, TX).

## Results

### Older adults adapt slower than younger adults

**Figure 2:**
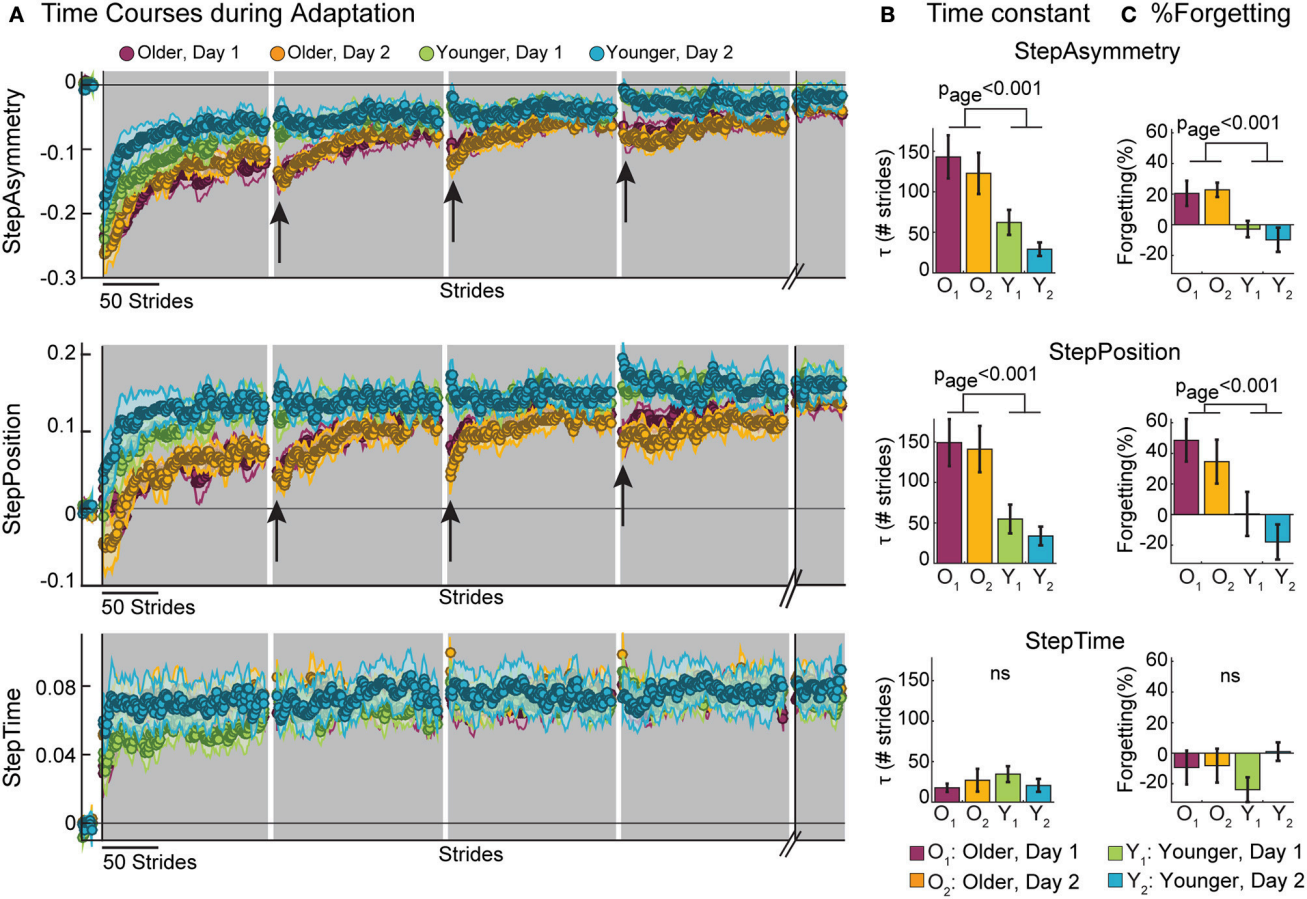
**Early Adaptation Behavior. (A)** Stride-by-stride time courses during baseline and adaptation for StepAsym, StepPosition, and StepTime are shown. Shaded gray areas represent the adaptation period. Resting breaks, when subjects were not walking, are indicated by the white regions in between shaded areas. The last 50 strides of the second adaptation block (before subjects walk overground) are also shown. Black arrows indicate the decays in adapted state due to the passage of time during the resting breaks. Colored dots represent the average of 5 consecutive strides and colored shaded regions indicate the standard error for each group. **(B)** Bar plots indicate the mean time constants (i.e., τ) per group ± standard errors and statistical difference lines between groups illustrate significant ANOVA effects of age. Remember that a large τ indicates that subjects slowly adapted. Note that, on average, subjects' time constants are <150 strides indicating that they occurred before the first resting break. **(C)** Bar plots indicate the mean %Forgetting per group ± standard errors and statistical difference lines between groups illustrate significant ANOVA effects of age.

We observed that older adults took more steps than young before reaching a plateau in their behavior during adaptation. Figure 2A (top panel) shows the time course of StepAsym during baseline and adaptation for all groups. Note that time courses for older adults during the first and second visits (Figure 2A top panel: purple and yellow curves, respectively) lay lower than those of younger subjects (Figure 2A top panel: green and blue curves). These differences between age groups are substantiated by the significant group effect on StepAsym time constants (Figure 2B), which characterized the rate at which subjects from each group adapted [*F*_(1, 35)_ = 17.14, *p* < 0.001]. In addition, we did not find an exposure effect on τ [*F*_(1, 35)_ = 1.60, *p* = 0.21], indicating that older adults adapted slower than young the first and second time they experienced the split-belt perturbation. In sum, older adults adapted their step asymmetry more slowly than younger adults regardless of exposure.

Moreover, the effect of age on adaptation dynamics was due to differences in the rate at which older vs. younger adults adjusted spatial, but not temporal aspects of step asymmetry. This is shown by the differences in time courses between StepPosition (Figure 2A middle panel) and StepTime (Figure 2A bottom panel). Note that there are differences across age groups in StepPosition, but all time courses overlap in StepTime. Consistently, we observed a significant age effect on time constant τ for StepPosition [*F*_(1, 35)_ = 18.37, *p* < 0.001], but not StepTime [*F*_(1, 35)_ = 0.57, *p* = 0.46] (Figure 2B). This difference in adaptation dynamics of spatial and temporal gait features was maintained across visits, as indicated by the lack of exposure effect on τ for StepPosition [*F*_(1, 35)_ = 0.38, *p* = 0.54] and StepTime [*F*_(1, 35)_ = 0.06, *p* = 0.81]. It is important to note that the differences in time constants, indicating the rate at which subjects adapted, were not driven by differences in the early behavior (i.e., average of the first 5 strides of adaptation) or differences in steady states; given that neither the early behavior [StepAsym: *F*_(1, 35)_ = 0.96, *p* = 0.33; StepPosition: *F*_(1, 35)_ = 0.19, *p* = 0.66] nor the steady states (reported in section “Older Adults Adapt and Learn as Much as Young”) were significantly different across age groups. Therefore, we found age-related differences that were independent of exposure in the adaptation rate of spatial and not temporal gait features.

### Older adults are more forgetful

Our results also indicate that the spatial pattern learned on the split-belt treadmill decays with the passage of time during the rest-breaks in older, but not in younger adults. One can observe in the time courses of StepAsym (Figure 2A top panel) and StepPosition (Figure 2A middle panel) value discrepancies before and after resting breaks in the behavior of older adults (indicated by black arrows). This decay was not observed in the traces of younger subjects, who maintain similar StepAsym and StepPosition values pre- and post- each break. On the other hand, we observed that StepTime values (Figure 2A bottom panel) did not decay during the rest-breaks. The decay of spatial motor memories was quantified by %Forgetting values shown in Figure 2C. We observed that the mean %Forgetting for old groups is significantly higher than for young groups in StepAsym [*F*_(1, 35)_ = 15.98, *p* < 0.001] and Step Position [*F*_(1, 35)_ = 13.20, *p* < 0.001], but not in StepTime [*F*_(1, 35)_ = 0.22, *p* = 0.64]. Moreover, these results were maintained with repeated exposures, as indicated by the non-significant exposure effect across parameters [StepAsym: *F*_(1, 35)_ = 0.11, *p* = 0.74, StepPosition: *F*_(1, 35)_ = 1.34, *p* = 0.26, and StepTime: *F*_(1, 35)_ = 1.80, *p* = 0.19]. Thus, %Forgetting of adapted spatial gait features in older adults was not reduced with repeated exposures of the locomotor paradigm. Taken together, these results show that older adults “forget” the adapted spatial pattern learned on the split-belt treadmill during rest-breaks, whereas they maintain the temporal one.

**Figure 3:**
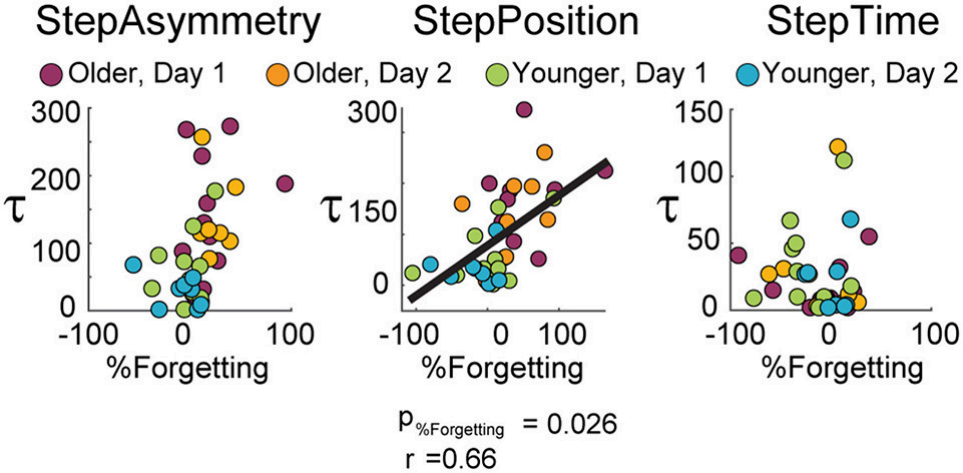
**Scatter plots illustrate the relationship between %Forgetting and the adaptation time constant (τ)**. Multiple regression analysis indicate %Forgetting was a significant predictor of the adaptation rate of StepPosition, but not StepAsym or StepTime.

Moreover, forgetting of spatial gait features predicted the rate at which subjects adapted. Figure 3 shows the results from the multiple regression analyses to determine the predictive power of age group, exposure, and %Forgetting on τ. We observed that %Forgetting was a significant predictor (*t* = 2.32, *p* = 0.026) of the adaptation rate of StepPosition, quantified by the time constant τ. The positive relation between these two measures indicated that as %Forgetting increased, subjects adapted slower–that is, they had a larger time constant τ (τ_*predicted*_ = 0.62 × *%Forgetting* − 4.40 × *exposure* + 68.47 × *age* + 56.88, *F*_(3, 34)_ = 8.84, *p* < 0.001, *r* = 0.66). Note that the relation between %Forgetting and τ was not observed in StepAsym (*t* = 1.12, *p* = 0.27) and StepTime (*t* = 0.09, *p* = 0.93). Thus, %Forgetting determined the adaptation rate of StepPosition for all age groups, but not of the other two parameters. It is worth mentioning that 6 out of 11 old subjects reached 63.2% of their spatial adapted state, which was used to quantify τ, after the first rest-break. Therefore, it might be possible that these subjects took longer to reach their steady state because of the decay in adapted spatial gait features occurring during the break. Lastly, age was a significant predictor of StepPosition (*t* = 2.66, *p* = 0.012) and StepAsym (*t* = 2.82, *p* = 0.008), but not StepTime (*t* = −0.75, *p* = 0.46) and exposure was not a predictor for any parameter (StepAsym, *t* = −1.20, *p* = 0.24; StepPosition, *t* = −0.19, *p* = 0.85; StepTime, *t* = −0.25, *p* = 0.81), which is consistent with results shown in Figure 2C. In conclusion, slower adaptation and forgetting of spatial motor memories were related in all age groups.

### Older adults adapt and learn as much as young

**Figure 4:**
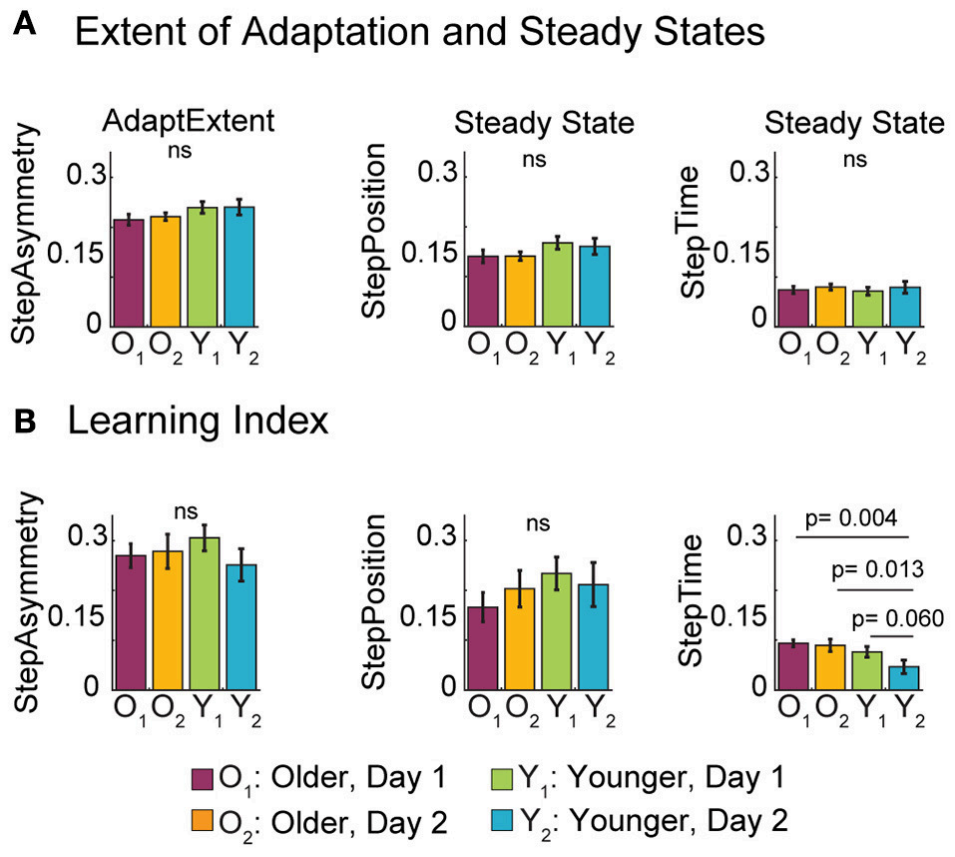
**Late Adaptation Behavior and Learning. (A)** Bar plots indicate the mean extent of adaptation (AdaptExtent) and adapted steady states per group ± standard errors. In general, all subjects reached the same adapted state. **(B)** Bar plots show the mean learning index per group ± standard errors. Recall that the learning index is quantified by the average after-effects on the treadmill during the catch trial, when both belts move at the same speed. We only found an age effect on the Learning Index for StepTime, which was driven by the smaller after-effects of young adults during their second visit compared to other groups (*post-hoc p*-values and statistical difference lines shown). We believe that this smaller after-effect indicates that young subjects can switch faster between the split and tied StepTime patterns during their second visit.

While older adults adjusted their gait more slowly, they eventually reached a similar adapted state as young. Note in the time courses shown in Figure 2A that young and old reached the same adapted state in all parameters at the end of the adaptation period during both visits. These similarities are indicated in Figure 4A by non-significant age [*F*_(1, 35)_ = 3.68, *p* = 0.063] and exposure effects [*F*_(1, 35)_ = 0.08, *p* = 0.78] on the AdaptExtent of StepAsym. Note that the strong trend of the age factor on the AdaptExtent was not observed when 1 (out of the 11) young subjects was removed from the analysis [*F*_(1, 34)_ = 1.55, *p* = 0.22]. Thus, while an outlier subject adapted more than the others, in general subjects from all age groups could counteract equally well the split-belt perturbation during both experimental visits. We also observed that young and old groups used similar adaptation strategies, as indicated by the same steady states reached in the adaptation of StepPosition [*F*_(1, 35)_ = 3.45, *p* = 0.072] and StepTime [*F*_(1, 35)_ = 0.04, *p* = 0.83] across age groups. Again, the strong trend of the age factor was driven by the behavior of the same subject reported above and was not observed if this subject was removed from the analysis [*F*_(1, 34)_ = 1.52, *p* = 0.23]. Thus, we concluded that age does not have an effect on adapted steady states even if one subject reached a larger adapted state in StepPosition. Lastly, these similarities in adapted states across age groups were maintained with exposure, as shown by the non-significant exposure effect on the steady state of StepPosition [*F*_(1, 35)_ = 0.06, *p* = 0.81] and StepTime [*F*_(1, 35)_ = 0.63, *p* = 0.43]. Therefore, neither age nor exposure changed the extent of adaptation or the motor strategy used to counteract the split-belt perturbation.

In addition, older adults were able to learn as much as young. Recall that learning was quantified with the magnitude of after-effects during a catch trial on the treadmill, when the split-belt perturbation was removed. Figure 4B indicates that after-effects on the treadmill were not affected by subjects' age. Accordingly, age did not have an effect on the learning index of StepAsym [*F*_(1, 35)_ = 0.10, *p* = 0.75] or StepPosition [*F*_(1, 35)_ = 1.49, *p* = 0.23]. Conversely, StepTime after-effects were significantly smaller for young than old subjects [*F*_(1, 35)_ = 6.82, *p* = 0.013]. We further observed that this age-related difference was driven by the smaller after-effects of younger adults during their second visit compared to all other groups (*post-hoc* analysis: young 2nd visit vs. old 1st visit: *t* = −3.07, *p* = 0.004; young 2nd visit vs. old 2nd visit: *t* = −2.62, *p* = 0.013; and young 2nd visit vs. young 1st visit: *t* = −1.95, *p* = 0.06). Therefore, while exposure did not have an effect in the learning index for any of the parameters [StepAsym: *F*_(1, 35)_ = 0.62, *p* = 0.43; StepPosition: *F*_(1, 35)_ = 0.04, *p* = 0.84; StepTime *F*_(1, 35)_ = 2.39, *p* = 0.13], younger adults had smaller after-effects on their second visit compared to the other groups. We believe these results indicate that younger adults could switch faster between the adapted split and tied patterns on their second visit, and not necessarily that they learned less. In sum, healthy aging does not impair the ability to adapt and store new sensorimotor representations of walking, but diminishes the ability to switch temporal stepping patterns on the treadmill based on prior experience.

### Older adults transfer more than younger adults

**Figure 5:**
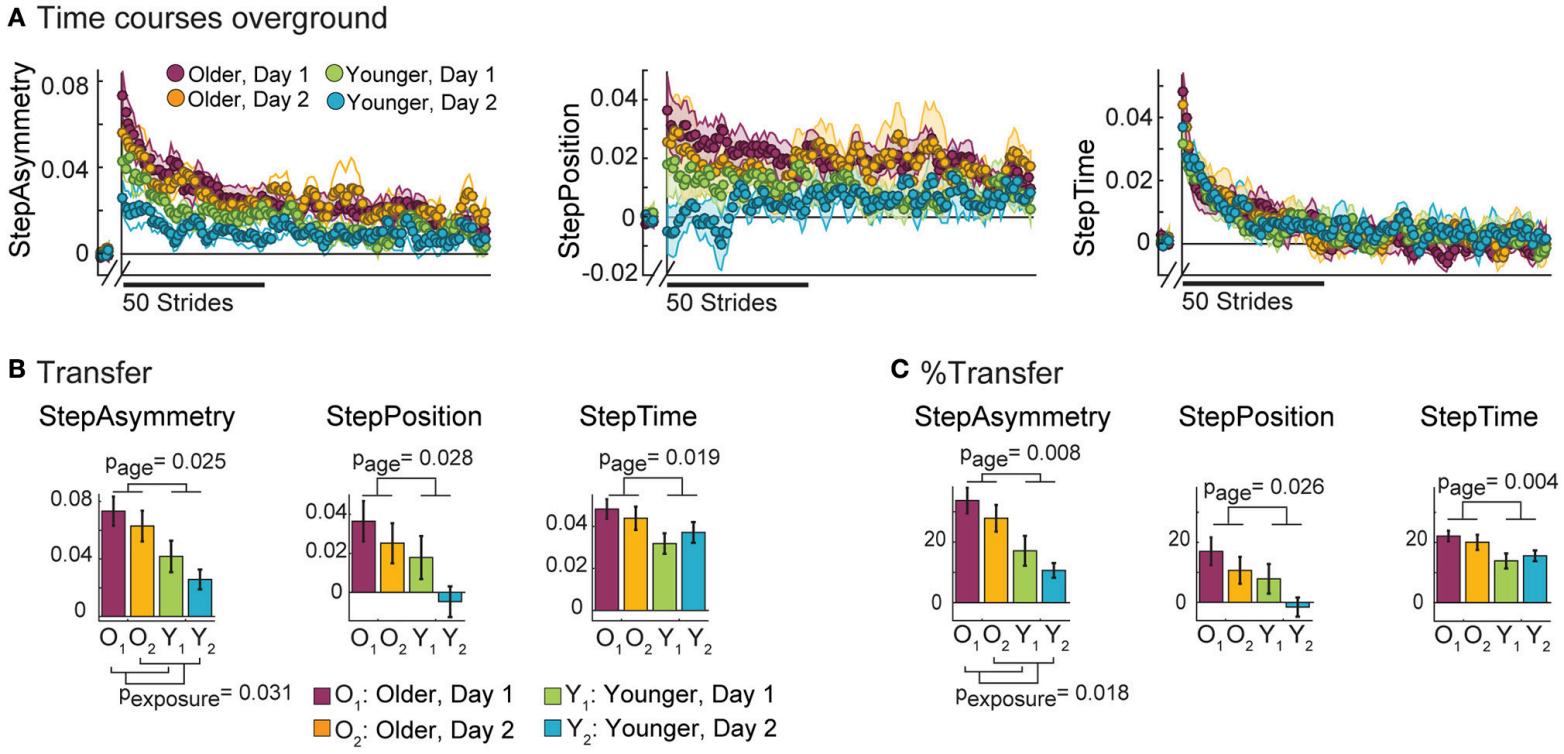
**Overground Behavior. (A)** Stride-by-stride time courses of StepAsym (left), StepPosition (middle), and StepTime (right) are shown for baseline and post-adaptation overground walking. Colored dots represent the average of 5 consecutive strides and colored shaded regions indicate the standard error for each group. **(B)** Bar plots indicate the mean Transfer values per group ± standard errors and statistical difference lines between groups illustrate significant ANOVA effects of age. These quantify the initial after-effects when walking overground right after split-belt walking. **(C)** Bar plots indicate the mean %Transfer values per group ± standard errors and statistical difference lines between groups illustrate significant ANOVA effects of age. %Transfer values indicate the amount of initial after-effects as a percent of AdaptExtent on the split-belt condition. In other words, %Transfer values takes into account how well subjects adapted their gait on the treadmill. While an age effect is found in all parameters, exposure effects are only found for StepAsymmetry, but not for StepPosition [Transfer: *F*_(1, 35)_ = 2.68, *p* = 0.11 and %Transfer: *F*_(1, 35)_ = 2.97, *p* = 0.094] or StepTime [Transfer: *F*_(1, 35)_ = 0.01, *p* = 0.93 and %Transfer: *F*_(1, 35)_ = 0.01, *p* = 0.92].

Older adults have difficulty switching movement patterns when transitioning from walking on the treadmill to walking overground. This was indicated by the larger after-effects in all parameters observed in older adults walking overground compared to young. Note in Figure 5A that time courses from older groups start from larger initial values compared to younger groups. Consistently, significant age effects on Transfer were found in all parameters (Figure 5B). Specifically, older adults transferred more than young in StepAsym [*F*_(1, 35)_ = 5.52, *p* = 0.025], StepPosition [*F*_(1, 35)_ = 5.23, *p* = 0.028], and StepTime [*F*_(1, 35)_ = 6.10, *p* = 0.019]. The same results were observed when Transfer was expressed as a percent of AdaptExtent [Figure 5C; StepAsym: *F*_(1, 35)_ = 7.95, *p* = 0.008, StepPosition: *F*_(1, 35)_ = 5.40, *p* = 0.026, StepTime: *F*_(1, 35)_ = 9.36, *p* = 0.004]. Moreover, we found that exposure had an effect on Transfer [*F*_(1, 35)_ = 5.05, *p* = 0.031] and %Transfer of StepAsym [*F*_(1, 35)_ = 6.22, *p* = 0.018], suggesting that subjects could better disengage the movements learned on the treadmill when walking overground during their second visit. However, *post-hoc* analysis revealed that this exposure effect was driven by differences only in younger subjects. Specifically, younger subjects had less overground after-effects in StepAsym on their second visit compared to their first one when quantified as Transfer (*t* = −1.99, *p* = 0.055) or %Transfer (*t* = −2.04, *p* = 0.049). On the other hand, older subjects did not have statistically different Transfer (*t* = −1.16, *p* = 0.26) or %Transfer (*t* = −1.45, *p* = 0.16) across visits. Thus, younger subjects were able to use the experience switching between walking contexts in their first visit to contextualize movements better during their second visit. Conversely, older adults transferred equally across visits. Taken together, our results indicate that older adults have diminished ability for switching movement patterns across walking situations and, unlike younger adults, this is not improved with prior experiences transitioning between walking contexts.

### Transfer in older adults is correlated with cognitive switching

**Figure 6:**
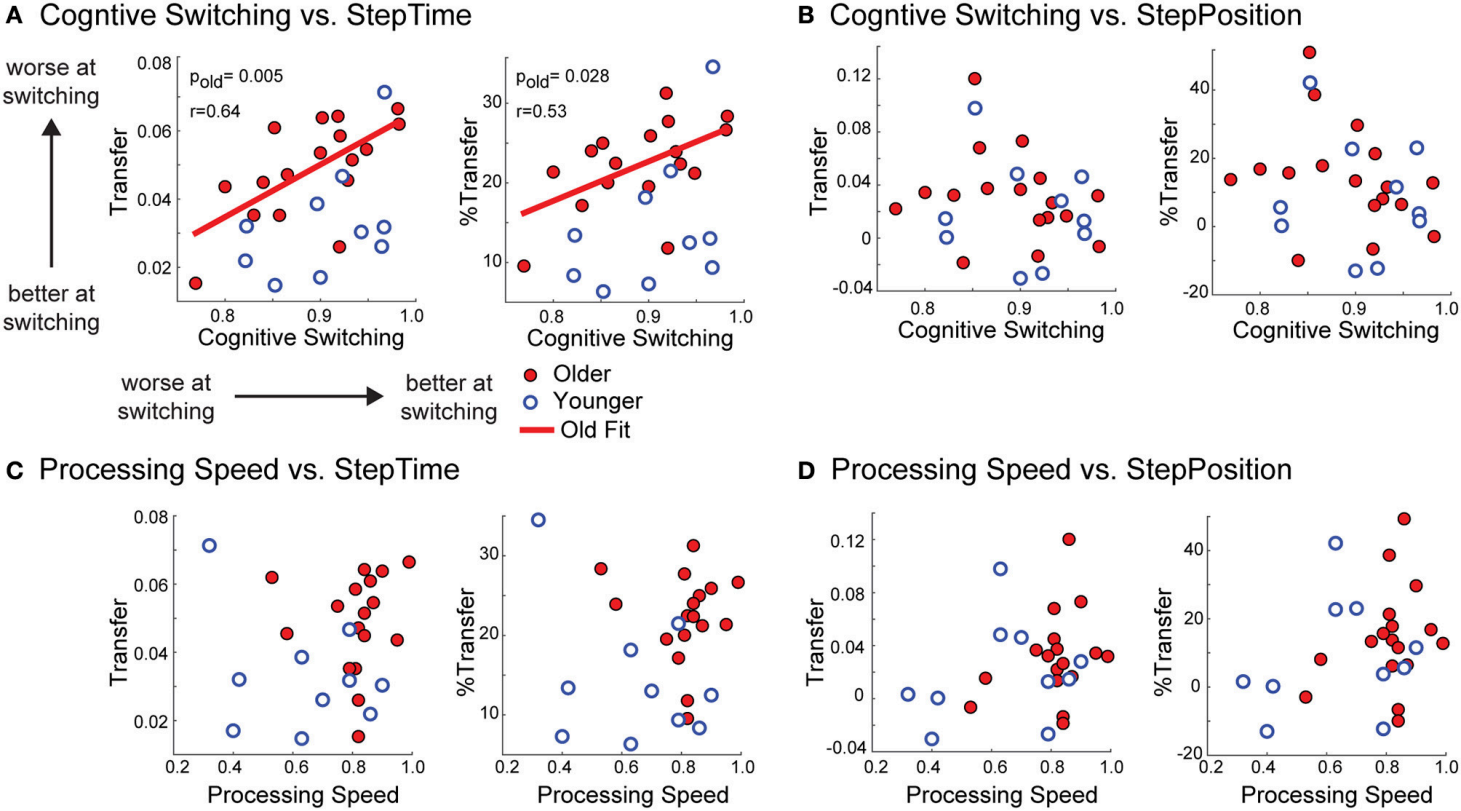
**(A–D)** Scatter plots of cognitive abilities vs. motor transfer. Scatter plots of cognitive ability vs. transfer. Each panel illustrates the scatter plots of cognitive abilities that we tested (i.e., cognitive switching and processing speed) vs. transfer and %Transfer of StepTime and StepPosition for younger and older subjects. A significant relation is only observed between old adults cognitive switching and transfer of StepTime after-effects when expressed as absolute values (Transfer) or as a percent of AdaptExtent on the treadmill (%Transfer). On the other hand, non-significant correlations were found between cognitive switching and Transfer of StepTime after-effects in young adults when quantified as Transfer [*F*_(1, 8)_ = 2.32, *p* = 0.17] or %Transfer [*F*_(1, 8)_ = 1.19, *p* = 0.20] of StepTime. Thus, motor and cognitive switching were only related in old, but not young subjects.

We observed that older subjects' performance in a cognitive switching task was a predictor of motor switching, which was quantified by movement transfer across walking contexts. Recall that large transfer values indicated that subjects were poor at switching walking patterns when transitioning from the treadmill to overground. Interestingly, cognitive switching was inversely related to motor switching (Figure 6A). In other words, old adults that were better at switching in the cognitive task were worse at switching locomotor patterns in the motor task. Note that this negative correlation was only true for motor switching in the temporal domain when expressed as Transfer [*F*_(1, 15)_ = 10.66, *p* = 0.005, *r* = 0.64, $\hat{Transfer}=0.15\times CognitiveSwitching-0.09$] or %Transfer [*F*_(1, 15)_ = 5.95, *p* = 0.028, *r* = 0.53, $\hat{\%Transfer}=49.62\times CognitiveSwitching\;-\;21.96$]. On the other hand, cognitive switching was not related to motor switching in the spatial domain [Figure 6B; Transfer: *F*_(1, 15)_ = 0.82, *p* = 0.38, %Transfer: *F*_(1, 15)_ = 1.40, *p* = 0.26]. In addition, motor and cognitive switching were only related in the performance of old, but not young subjects (Figure 6A). This is shown by the non-significant correlation between cognitive switching and motor switching in young adults when quantified as Transfer of StepTime [*F*_(1, 8)_ = 2.32, *p* = 0.17] and StepPosition [*F*_(1, 8)_ = 0.13, *p* = 0.73] or %Transfer of StepTime [*F*_(1, 8)_ = 1.19, *p* = 0.20] and StepPosition [*F*_(1, 8)_ = 0.10, *p* = 0.75]. Importantly, this relation between cognition and motor performance was specific to cognitive switching, and not to older adults' cognitive performance in general (Figures 6C,D). This can be observed by the lack of relation between processing speed and motor switching in young and old subjects when expressed as Transfer of StepTime [old group: *F*_(1, 15)_ = 0.06, *p* = 0.81, young group: *F*_(1, 8)_ = 0.93, *p* = 0.36] and StepPosition [old group: *F*_(1, 15)_ = 1.54, *p* = 0.23; young group: *F*_(1, 8)_ = 0.39, *p* = 0.55] or %Transfer of StepTime [old group: *F*_(1, 15)_ = 0.06, *p* = 0.80; young group: *F*_(1, 8)_ = 1.43, *p* = 0.27] and StepPosition [old group: *F*_(1, 15)_ = 1.25, *p* = 0.28; young group: *F*_(1, 8)_ = 0.30, *p* = 0.60]. In sum, cognitive switching in older adults interfered with motor switching across walking contexts of temporal gait features.

### Older and young adults have similar remaining after-effects when returning to the training context

**Figure 7:**
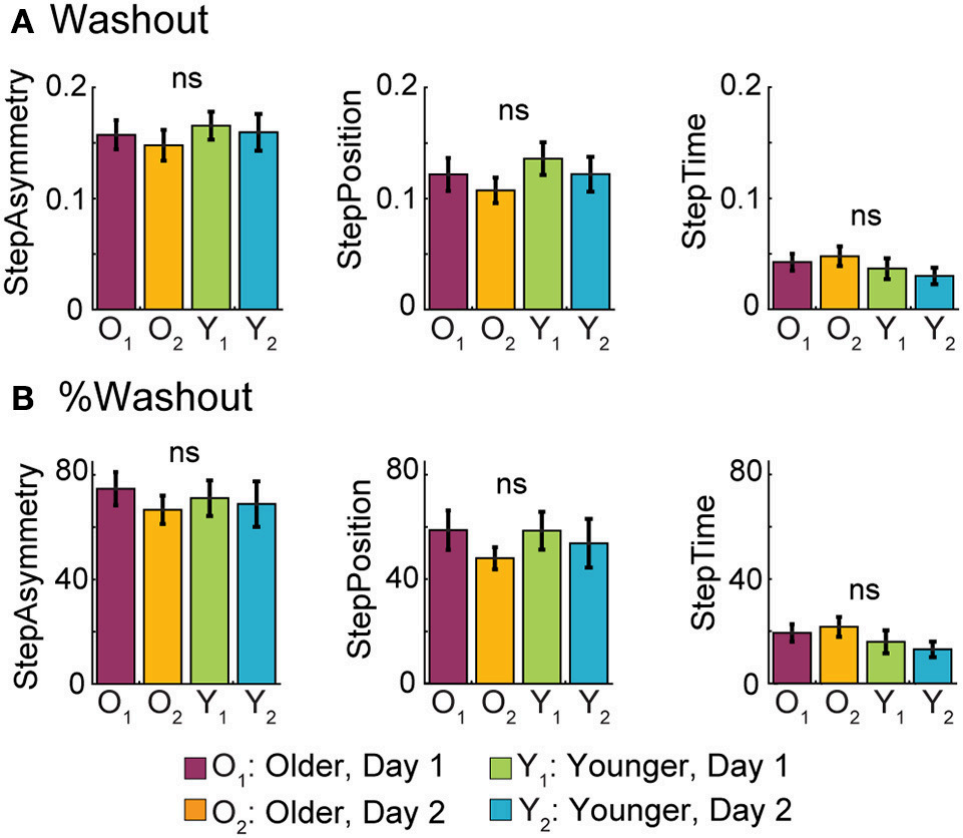
**Washout of Split-belt After-effects Following Overground Walking. (A)** Bar plots indicate the mean Washout values per group ± standard errors. These quantify the initial after-effects when returning to the treadmill after overground walking. **(B)** Bar plots indicate the mean %Washout values per group ± standard errors. %Washout values indicate the amount of remaining after-effects on the treadmill as a percent of AdaptExtent during the split-belt condition. In other words, %Washout values takes into account how well subjects adapted their gait on the treadmill. %Washout values of 100% indicate that the adapted movements on the treadmill remain intact after the overground walking experience.

Neither age nor exposure affected the magnitude of after-effects when returning to the treadmill following overground walking. In Figure 7A it can be seen that all groups had similar remaining after-effects when returning to walk on the treadmill after overground walking. Thus, age did not have an effect on Washout values for StepAsym [*F*_(1, 35)_ = 0.50, *p* = 0.48], StepPosition [*F*_(1, 35)_ = 0.99, *p* = 0.33], or StepTime [*F*_(1, 35)_ = 1.62, *p* = 0.21]. Furthermore, these similarities between groups were maintained across visits, as shown by the non-significant exposure effect on Washout values of StepAsym [*F*_(1, 35)_ = 0.31, *p* = 0.58], StepPosition [*F*_(1, 35)_ = 0.94, *p* = 0.34], and StepTime [*F*_(1, 35)_ = 0.01, *p* = 0.94]. Similar results were obtained when remaining after-effects on the treadmill were expressed as a percent of the extent of adaptation (AdaptExtent) on the treadmill (Figure 7B). Specifically, age group did not affect %Washout for StepAsym [*F*_(1, 35)_ = 0.03, *p* = 0.87], StepPosition [*F*_(1, 35)_ = 0.10, *p* = 0.76], or StepTime [*F*_(1, 35)_ = 2.19, *p* = 0.15]. Additionally, exposure did not affect %Washout for StepAsym [*F*_(1, 35)_ = 0.56, *p* = 0.46], StepPosition [*F*_(1, 35)_ = 1.09, *p* = 0.30], or StepTime [*F*_(1, 35)_ = 0.01, *p* = 0.94]. Therefore, motor memories specific to the treadmill were not washed out by overground walking, regardless of subjects' age or prior experience transitioning between these two walking contexts.

## Discussion

We investigated how healthy aging affects one's ability to adapt, learn, retain, and switch locomotor patterns across walking contexts and how cognitive action selection impacted motor switching. We found that healthy aging does not alter sensorimotor adaptation, but has a negative impact on the specificity and retention of motor memories acquired during novel situations. Interestingly, cognitive and motor switching were inversely related in older adults. Thus, cognitive action selection hindered, rather than compensated for age-related deficits in motor specificity.

### Older adults can adapt their gait and learn new walking patterns

We found that healthy aging does not limit the ability to adapt walking movements in response to sustained changes in the environment. This was indicated by the similarity in adapted behaviors across age groups, as reported before (Malone and Bastian, 2016). This finding is at odds with previous motor adaptation studies showing limitations in the adapted state reached by old participants in walking (Bruijn et al., 2012) and reaching (e.g., McNay and Willingham, 1998; Seidler, 2006; Hegele and Heuer, 2010, 2013; Langan and Seidler, 2011; Huang and Ahmed, 2014). These distinct findings can be explained by the higher instances of large errors occurring after resting breaks, which were present in our protocol unlike the other studies. Notably, it has been shown that learning is facilitated if errors are large (Körding and Wolpert, 2004; Kluzik et al., 2008; Wei and Körding, 2009; Schweighofer et al., 2011; Torres-Oviedo and Bastian, 2012; Pauwels et al., 2015) and consistent (Korenberg and Ghahramani, 2002; Burge et al., 2008; Wei and Körding, 2010; Castro et al., 2014). Therefore, old adults can adapt their gait to the same degree as young when they experience multiple instances of large and consistent errors.

Our results also show that healthy aging does not impair the ability to learn new representations of environment dynamics. We observed that after-effects, which result from adapted and stored representations of the environment, are equally large in old and young subjects before or after the overground walking. This observation is consistent with previous studies showing that sensorimotor recalibration upon external perturbations is not impaired in older adults for walking (Bruijn et al., 2012) and reaching behaviors (e.g., Buch et al., 2003; Bock, 2005; Bock and Girgenrath, 2006; Seidler, 2007). Thus, older adults can acquire new sensorimotor representations of the world through interactions with the environment.

### Older adults are “resistant” to updating movements according to the context

While older adults can adapt and learn new movements as well as young, they are resistant to updating their movements. This claim is supported by (1) older adults' slower adaptation rate and (2) their difficulties switching walking patterns according to the context. Both of these findings are consistent with other work showing slower adaptation in older adults (e.g., Rodrigue et al., 2005; Anguera et al., 2012; Bruijn et al., 2012; Trewartha et al., 2014) and larger carry-over of movements across conditions in reaching adaptation (e.g., Fernández-Ruiz et al., 2000; Bock and Girgenrath, 2006; Heuer and Hegele, 2008). Thus, healthy aging reduces the ability to update motor commands according to changes in the environment.

The resistance to updating movements in older adults indicates higher reliance on previous experiences, which could be explained by either (1) poorer sensitivity to errors or (2) higher costs associated to exploration in older adults. Consider that healthy aging increases sensory (Zhang et al., 2008; Goble et al., 2009; Maheu et al., 2015) and motor noise (Holloszy and Larsson, 1995; Laidlaw et al., 2000; Kallio et al., 2012; Vanden Noven et al., 2014), which reduce the certainty of sensed errors updating internal representations of the environment (Wolpert et al., 1995). Consequently, the sensitivity to errors decreases, increasing the reliance on prior estimations of the environment. This idea is further supported by findings indicating that aging impacts the integrity of the cerebellum (Luft et al., 1999; Raz et al., 2005), which regulates the sensitivity to errors driving motor adaptation (Criscimagna-Hemminger et al., 2010). Alternatively, older adults are in general risk averse (Albert and Duffy, 2012; Tymula et al., 2013) and risk-sensitivity has been shown to influence sensorimotor control (Nagengast et al., 2010; O'Brien and Ahmed, 2015) and motor adaptation (Trent and Ahmed, 2013). Thus, it is possible that the aged motor system exploits prior experiences rather than exploring new movements to avoid risks such as falling, which have more serious consequences in old than young populations (Talbot et al., 2005; Mitchell et al., 2010). In sum, older adults need to accumulate a lot of evidence in a new environment before updating their movements possibly due to large sensory and motor noise or their fear of risks associated to movement exploration.

### Cognition interferes with motor switching in older adults

We observed an unexpected inverse correlation between cognitive and motor switching, which might be explained by the recruitment of cognitive centers compensating for age-related basal ganglia deficits controlling motor switching. It has been shown that the basal ganglia mediates both cognitive (Dreher and Grafman, 2002) and motor switching (Brown and Almeida, 2011; Leunissen et al., 2013; Balser et al., 2014). It has also been shown that cognitive centers are recruited in switching tasks performed by older adults (Coxon et al., 2010) to compensate for age-related functional deficits in the basal ganglia (Bäckman et al., 2006; Ota et al., 2006). However, cognitive compensation can worsen the performance of implicitly controlled tasks (Boyd and Winstein, 2004). Thus, we conclude that the recruitment of cognitive resources for switching augments motor switching deficits in older adults instead of effectively correcting them. This idea is supported by evidence showing that cognitive and motor switching are inversely related in Parkinson patients (Inzelberg et al., 2001) exhibiting stronger functional deficits in the basal ganglia than unimpaired old subjects. Taken together, our findings indicate that utilizing cognitive resources interferes with motor switching instead of compensating for basal ganglia related motor switching deficits in older adults.

### Older adults encode motor memories susceptible to the passage of time

Our results show that aging affects the retention of movements since older adults exhibit forgetting during resting breaks and naïve-like behavior after repeated exposure of the locomotor task. These observations are consistent with other studies showing forgetting during breaks of newly acquired walking patterns (Malone and Bastian, 2016) and lack of savings in older populations (Bierbaum et al., 2011). Previous work has shown that patients with cerebellar damage also forget during sitting breaks (Izawa et al., 2012), suggesting that age-related cerebellar degradation (Luft et al., 1999; Raz et al., 2005) might underlie the observed forgetting in older adults. Additionally, decays of adapted movements might also stem from structural decline of the motor cortex (Fjell and Walhovd, 2010), which mediates the resilience of adapted movements (Galea et al., 2011). Lastly, it has been proposed that forgetting is mediated by fast adaptation processes (Izawa et al., 2012), which learn fast and forget fast (Smith et al., 2006). However, this interpretation is not supported by our results considering that subjects who forgot the most were those who adapted the slowest, and thereby relied more in slow rather than fast adaptation processes. Of note, older adults participating in our study systematically took longer sitting breaks than young subjects. Thus, further studies are needed to determine the impact of the duration of the breaks on the extent of forgetting in old and young populations. In sum, degradation of cerebellum and motor cortex in older adults might explain forgetting of adapted movements observed in older populations.

### Differential aging effect on spatial and temporal control of the limbs

Age-related deficits in adaptation rate, motor switching, and retention predominantly affect spatial gait features while temporal gait features remained largely intact. This robust adaptation in the temporal domain has also been observed in children and in patients with cerebellar (Vasudevan et al., 2011) or cerebral lesions (Tyrell et al., 2014). The control of temporal features might be more robust to aging and brain lesions because of its impact on gait stability. Note that failure to adapt temporal aspects of gait to match environmental demands would lead to falling, whereas poor adaptation of spatial gait features decreases gait efficiency (Finley et al., 2013). While future work is needed to determine the neural correlates mediating the distinct adaptation and generalization of spatial and temporal gait features, our results indicate that those underlying the temporal control of the limb are more resilient to healthy aging.

### Clinical implications

Retention and transfer are clinically relevant aspects of motor learning and our findings suggest that increasing one will not necessarily increase the other when training older populations. This is an interesting finding considering that previous behavioral work suggested that retention and transfer were positively correlated. Specifically, adaptation paradigms that increased retention also increased transfer (Klassen et al., 2005; Huang and Shadmehr, 2009) suggesting a positive relation between these variables. Our results showing a negative relation between retention and transfer in older adults indicate that they should be targeted separately when training old populations.

Finally, the negative relation between cognitive and motor switching has significant implications into fall prevention therapies. Note that age-related deficits in switching walking patterns could contribute to older adults' propensity to falling. We find that cognitive switching interferes with implicit mechanisms controlling motor switching in older adults. Therefore, fall prevention interventions should focus on recruiting the remaining implicit switching mechanisms, rather than the use of explicit switching strategies.

## Conclusion

In conclusion, we found that healthy aging does not alter sensorimotor adaptation, but impairs the motor systems' ability to retain and switch motor patterns according to the context. Poor motor switching in older populations might be compensated by recruiting cognitive resources. However, this cognitive mediated compensation interferes with the remaining implicit control of motor switching. Our results are significant because they provide knowledge on how cognition influences motor control in older populations, which could be used to develop more effective treatments for age-related mobility impairments. Specifically, our findings suggest that reinforcing implicit mechanisms for motor switching would be a more effective approach for action selection in older adults than using cognitive strategies.

## Ethics statement

This study was carried out in accordance with the recommendations from the University of Pittsburgh Institutional Review Board with written informed consent from all subjects. All subjects gave written informed consent in accordance with the Declaration of Helsinki.

## Author contributions

CS contributions include acquisition, analysis, and interpretation of the data, drafting the work, final approval of the version to be published, and agreement to be accountable for all aspects of the work. HH contributions include acquisition and analysis of the data, revising work for important intellectual content, final approval of the version to be published, and agreement to be accountable for all aspects of the work. PS contributions include design of the work, revising work for important intellectual content, final approval of the version to be published, and agreement to be accountable for all aspects of the work. GT contributions include conception and design of the work, revising the work, final approval of the version to be published, and agreement to be accountable for all aspects of the work.

## Funding

CS is funded by a fellowship from the National Science Foundation (NSF-GRFP). This work was supported by a grant from the Pittsburgh Claude Pepper Older Americans Independence Center (P03 AG024827).

### Conflict of interest statement

The authors declare that the research was conducted in the absence of any commercial or financial relationships that could be construed as a potential conflict of interest.
